# An Unusual Case of Giant Abdominal Aortic Aneurysm Due to Type II Endoleak Persistent Failure

**DOI:** 10.7759/cureus.26300

**Published:** 2022-06-24

**Authors:** Catherine A Ostos Perez, Kristina D Menchaca, Erika A Ostos, Shaun Isaac

**Affiliations:** 1 Internal Medicine, University of Miami/John F. Kennedy (JFK) Medical Center, Atlantis, USA; 2 Biological Sciences, St. Petersburg College, St. Petersburg, USA; 3 Internal Medicine, John F. Kennedy (JFK) Medical Center, Atlantis, USA

**Keywords:** refractory shock, multifactorial shock, giant abdominal aortic aneurysm, endoleak repair, type ii endoleak, leaking aortic abdominal aneurysm, management of abdominal aortic aneurysms, abdominal aortic aneurysms

## Abstract

Abdominal aortic aneurysms (AAA) are considered giant when they exceed >10cm, and they are rare, with only a few cases described as >14cm. AAAs can be repaired through endovascular aneurysm repair (EVAR) or open surgery. EVAR involves the placement of a graft that contacts the aortic wall and the iliac vessels to prevent the aneurysm sac to have blood flow and pressure. One of the complications of EVAR is endoleak, the most common being type II. We describe an uncommon case of a progressively giant AAA with type II endoleak with poor evolution despite multiple repair attempts.

## Introduction

Abdominal aortic aneurysms (AAA) are considered large when they exceed >6cm, and giant when they exceed >10cm in the maximum transverse diameter [1]. Giant AAAs are rare, with only a very few cases described as larger than 14cm [2]. Thoracic and abdominal AAAs can be successfully repaired through endovascular aneurysm repair (EVAR) or open surgery [3,4]. EVAR involves the placement of graft that contact the aortic wall and the iliac vessels to prevent the aneurysm sac have blood flow and pressure, thus preventing its expansion and rupture. One of the complications of EVAR is endoleak, the most common being type II [4]. Type II endoleak usually undergoes surveillance unless there is sac expansion [5]. We describe an uncommon case of a progressively giant AAA with type II endoleak with poor evolution despite multiple endoleak repair attempts.

## Case presentation

**Figure 1:**
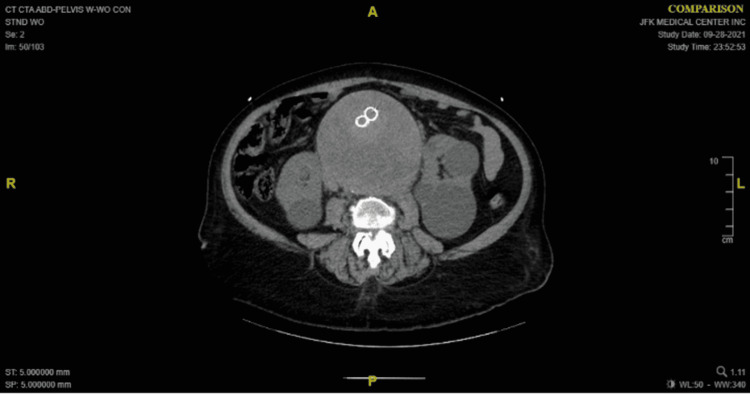
Axial view of the computerized tomography showing the AAA with the graft in place AAA: abdominal aortic aneurysms

**Figure 2:**
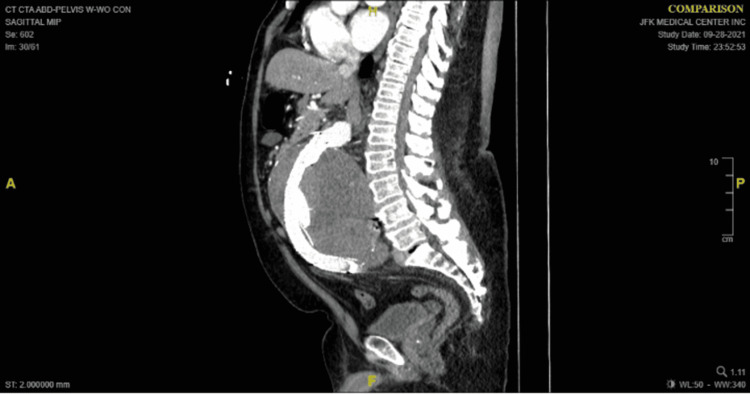
Sagittal view of the computerized tomography showing the graft and the large aneurysm around it

A 72-year-old male presented to the ER due to acute abdominal pain, nausea, and vomiting. He had a past medical history of hypertension, hyperlipidemia, obesity, and a 9cm abdominal aortic aneurysm (AAA) diagnosed four years prior to this admission. He underwent EVAR at that time, which was complicated by a type II endoleak with multiple attempts to stop it prior to this admission. He was found hypertensive (systolic blood pressure was on 145-200s) tender, obese distended abdomen, with a large midline palpable mass. Computerized tomography (CT) angiography revealed AAA with graft in place, increased since prior examination 14cm superior-inferior 13.3cm anteroposterior, 14cm transverse, with aneurysmal dilatation of the iliacs (4.6cm on right and 4.7cm on the left) and multiple renal cysts. Radiopaque foreign bodies within the large aortic aneurysm were unchanged (Figures 1, 2).

Upon admission, blood pressure was difficult to control and was transferred to ICU for close management. He was eventually diagnosed with *Streptococcus infantarius* bacteremia and started on antibiotics. The patient’s complex anatomy warranted him to have an open repair. Due to ongoing bacteremia, surgery was held off. During the following days, the patient developed worsening acute kidney failure and acidosis, requiring dialysis, related to worsening aneurysm size and perfusion. On day five of admission, an attempt for surgery was done; however during induction, the patient had coffee ground emesis with aspiration, was intubated, and the procedure was held. The patient developed multifactorial shock with multiple pressors, difficult to manage ventilation, and underwent cardiac arrest on day 10 of admission; return of spontaneous circulation was achieved after cardiopulmonary resuscitation (CPR) and one dose of epinephrine without rupture of the aneurysm. A second attempt for surgery was planned; however, the patient’s clinical status continued worsening. The patient’s family opted for hospice and expired shortly after.

## Discussion

Giant AAAs are a rare condition that can present with abdominal pain, pulsatile mass, or when ruptured with signs of hemorrhagic shock. EVAR has been demonstrated to be a treatment that decreases complications perioperatively, but in the long-term, the survival outcome compared with an open repair is not very different between both techniques [1].

One of the complications of EVAR is endoleak, the presence of blood flow towards the aortic sac after graft placement. There are five types of endoleak - type I and III involve flow from the proximal or distal end or from disconnection from the graft edges, type II is the most common, involving flow from visceral vessels that drain into the sac, type IV is from porous graft, and type V is undefined [4]. For type II, the management can be more conservative with surveillance and repairing only if there is noticeable growth of the aneurysmal sac. The repair of type II endoleak involves coil embolization of the blood vessels that are feeding the aneurysmal sac. Different imaging methods like CT, ultrasound (US), and magnetic resonance imaging (MRI) have been described for surveillance [5]; however, there is no clear consensus regarding how often the surveillance [6], and the benefit of early intervention vs continuous surveillance [2]. There are different studies that incline toward either approach. Thus, the recommendation is to assess risks and benefits and repair where there is an increase in aneurysmal sac [6].

In our case, the patient had a large AAA that became giant after multiple endoleak type II repair attempts of the aneurysmal sac during the years of follow-up. Due to the symptoms and significant enlargement, another EVAR repair was considered at first; however, due to the patient’s proximity to renal arteries, size mismatch, and bilateral iliac aneurysm, it was determined that he warranted open repair. This could not happen due to bacteremia, shock, and multiorgan failure in the setting of sepsis and AAA complications without any frank rupture (even after CPR). It is well-documented that type II endoleak sac enlargement rarely develops into acute rupture but may have consequences in the long term [4]. Therefore, some studies suggest the early intervention of type II endoleak [7]; however, there is not enough evidence to support this.

Different factors have been related to endoleak persistence including older age, absence of chronic obstructive pulmonary disease (COPD), and graft size, however, aneurysmal size is not one of them [4]. EVAR is a commonly used treatment for AAA, which has demonstrated superiority regarding invasiveness and immediate complications [7]. More studies are needed to determine the long-term survival and recurrence of endoleak and the need for repair vs surveillance.

## Conclusions

Management of AAA with complex anatomy in the setting of worsening symptoms and even shock requires a multidisciplinary approach and even then the outcome can be grim. Type II endoleak appears to be a marker of EVAR failure that is difficult to predict and treat effectively. It is still a gray area lacking enough evidence to support a specific time for action to prevent enlargement, thus the recommendation is surveillance and treatment when there are indications such as enlargement of the sac.

Our case showed that even after EVAR and endoleak type II repair, the prognosis of giant AAA is still poor. The patient already had a history of endoleak with attempted repair, which was complicated in this admission due to ongoing endoleak with the development of bacteremia, causing a multifactorial shock. Thus, the decision was made to hold further attempts at repair. Techniques such as EVAR have improved the immediate mortality and complications vs open AAA repair; however, more research is required to address the long-term differences for giant AAA. In cases like the one presented, the outcome can be unpredictable, thus it is important to closely follow-up and manage other risk factors to prevent further complications.
